# Developing a Method to Connect Thermal Physiology in Animals and Plants to the Design of Energy Efficient Buildings

**DOI:** 10.3390/biomimetics7020067

**Published:** 2022-05-24

**Authors:** Negin Imani, Brenda Vale

**Affiliations:** Wellington Faculty of Architecture and Design Innovation, Victoria University of Wellington, Wellington 6140, New Zealand; brenda.vale@vuw.ac.nz

**Keywords:** biomimetic design, sustainable design, biomimicry, thermal adaptation, thermoregulation

## Abstract

The literature shows that translating the thermal adaptation mechanisms of biological organisms to building design solutions can improve energy performance. In the context of bio-inspired thermoregulation several worthwhile attempts have been made to develop a framework for finding relevant thermal adaptation mechanisms in nature as inspiration for architectural design. However, almost all of these have followed a solution-based approach despite the problem-solving nature of architectural design. Given this, this research set out to take a problem-based approach to biomimetic design. The aim was to investigate the most effective way of accessing biological thermoregulatory solutions to assist architects in finding relevant biological inspirations for the thermal design of buildings. This required the development of an optimal structure for categorizing thermoregulatory mechanisms that could then be used as part of a framework for finding appropriate mechanisms for a particular architectural design problem. This development began with a three-step literature review to find, study, generalize and categorize a comprehensive list of thermal adaptation mechanisms used by animals and plants. This article describes how this literature review was carried out leading to the identification of nine main themes which were analysed for their practicality in informing the structure of the proposed framework. The selected themes were built around the common aspects of biology and architecture, and hence facilitated the categorization of biological thermoregulation mechanisms. This article thus explains the steps taken to develop a structure for generalizing and categorizing thermal adaptation strategies in nature. This article does not report on the list of thermal adaptation mechanisms identified in step 2 of the literature review. Instead, it presents the literature review workflow with a focus on step 3. Given that, discussion of the thermal adaptation mechanisms falls outside the scope of this article.

## 1. Introduction

Architectural researchers are already turning to the solutions used by biological organism to adapt to their thermal environment in the hope these might lead to sustainable buildings [1]. Biomimicry has also been recognised as a way of achieving energy efficient design [2,3,4]. In the construction industry biomimicry has the potential to reduce greenhouse gas emissions because nature uses low energy processes [5] and this suggests there might be numerous examples of biological organisms which could be explored for the energy efficient processes they use as a means of solving human design problems.

There are two different approaches to bio-inspired design, these being bottom-up (solution-based) and top-down (problem-based), which have also been called ‘biology push’ and ‘technology pull’ respectively [6]. Using the former approach designers have the biological knowledge, and hence the solution, at the outset [7], while the latter begins with a design problem as the basis for exploration of the natural world to look for solutions.

Because biology and architecture are two different knowledge domains with no obvious overlap, for bio-inspired design (BID) to be successful there needs to be a way for designers to find the relevant analogies in biology and subsequently translate these into architectural design principles. As no-one has investigated the possibility of developing a bio-inspired design framework specifically focusing on thermal adaption mechanisms for energy efficient buildings [8], this research, of which this literature review is a part, aimed to fill this gap.

In a problem-based approach the bio-inspired design process begins with design challenges, which in turn need to be connected to solutions offered by biological organisms in a systematic way, in a process known as design by analogy [9,10]. Given energy use reduction is a building design problem, a problem-based approach would seem to provide a better response to bio-inspired energy efficient building design (BEEBD). Problem-based BID has been researched in engineering fields [7,11] but not yet fully investigated in architectural design. Examples of frameworks that enable problem based bio-inspired design in other fields are: ‘Biomimicry 3.8′ led by Benyus, ‘BioTriz’ led by Julian Vincent [7], ‘Biomimetic for Innovation and Design Laboratory’ led by Li Shu [11], ‘Design and Intelligence Laboratory’ led by Goel and ‘Plants Biomimetic group’ led by Thomas Speck [12]. However, many of these efforts suffer from a lack of clarification in how to explore natural examples. One that is more detailed in how the investigation could be carried out is the *State-Action-Part-Phenomenon-Input-oRgan-Effect (SAPPhIRE)* model developed by Chakrabarti, Sarkar [13]. It contains several levels of abstraction of function through which the function of any biological system can be described. This framework also provides information about previously used biomimetic samples, and their structural, behavioural and functional aspects. In this software, textual representation of organisms is used as nouns, verbs and adjectives to describe engineering design problems. Biological engineering design is then achieved by matching the texts to the database. Mechanical engineers work with *SAPPhIRE* [14]. This model has been further examined by Sartori, Pal [15] by focussing on biological transfer mechanisms, and thus translating biological principles into design principles.

As shown in the *SAPPhIRE* example, knowledge is transferred from one domain to another through a link that is normally the abstraction of the functions, behaviours and processes of biological organisms. This was what the third step of the literature review described in this article was aiming to achieve. The intention was to examine the physiological functions and then match these to architectural processes used in designing energy efficient buildings. For each thermoregulatory solution, the functions, behaviours and processes were studied to allow for the translation of thermoregulatory principles from biology to architecture.

One architectural example of the problem-based approach is the framework developed by Badarnah (2012), although this was incomplete. Badarnah stressed the necessity of developing an optimal classification scheme accepting the fact that there could be various ways of categorising biological information, but did not do this, a goal that was addressed by this research, and which resulted in the development of the ThBA [8,16]. This is a tool that allows architects to find biological solutions relevant to the thermal problems in the design of a building.

Developing the ThBA required studying biology to find, classify and generalise the thermal regulation strategies used by living organisms. The intention was the ThBA would have two sides relating to the distinctive fields of biology and architecture with links between. These links would become the points through which the analogies identified from the natural world and their corresponding thermal adaptation principles could be transferred.

**Figure 1:**
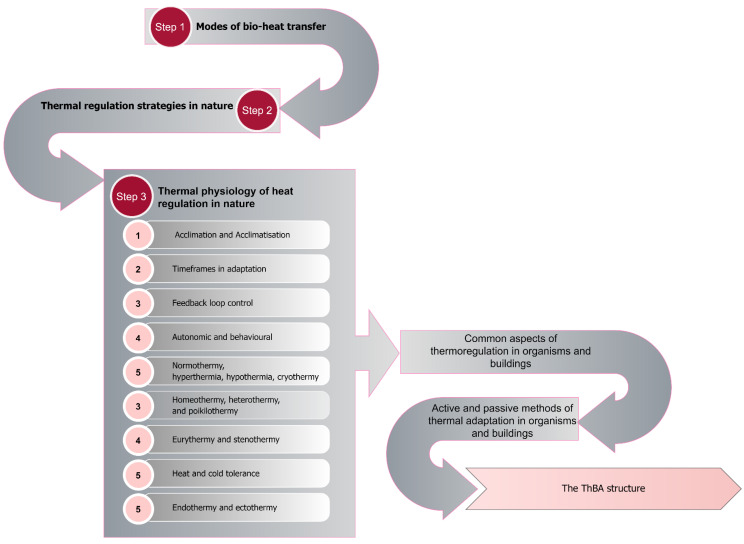
Steps in the literature review and their contribution to creating the ThBA.

As noted earlier, developing the ThBA began with a three-step literature review. The first step involved studying methods of heat transfer found in nature. Step two comprised a comprehensive literature review on the behavioural, physiological and morphological thermal adaptation strategies used by biological organisms. However, neither stage suggested a structure for categorising or generalising the thermoregulatory strategies, which would be a vital step in creating the ThBA. Step three involved investigating the key themes of thermal physiology as suggested in the original glossary of thermal physiology revised by the Commission for Thermal Physiology of the International Union of Physiological Sciences (IUPS Thermal Commission, 1987) (Figure 1). Full explanation of the testing and final development of the ThBA can be found elsewhere [8].

## 2. Materials and Methods

In steps one and two, relevant literature was investigated by searching appropriate data bases using key words. Sufficient articles were examined such that key themes emerged. For step one, the methods of heat transfer are well known, as shown below. The same approach was adopted for step two but when that failed to produce a way of categorizing thermal strategies a different approach was used for step three, which only used the glossary of thermal physiology. The original glossary has been modified following numerous comments from experts during its development. This research used Version 3 of the IUPS which contains 479 terms. These were examined and grouped in ways that had parallels with the thermal behaviour of buildings, as described below.

### 2.1. Step 1: Modes of Bio-Heat Transfer

Dry heat exchange or sensible heat transfer [17] normally occurs in three ways through transfer via conduction, convection and radiation.

The rate and direction at which heat transfers through conduction is dependent on the temperature difference between an animal’s body and the surface with which it is in contact. Some species of large mammals use behaviour to control conductive heat transfer by changing the temperature difference. For example, Chacma baboons (*Papio hamadryas ursinus*) in the Namib Desert do this by sand bathing [18]. This reduces their body temperature by facilitating conductive heat transfer to the cooler sand beneath the surface.

Convective heat transfer in contrast depends on the air temperature of the microclimate where an animal live [19,20,21]. Convective heat exchange is ‘forced’ when there is a wind and is ‘free’ in the absence of wind. An animal running quickly will force convective heat transfer but at the same time, heat is generated by muscular activity, so running may not be a way of cooling the body.

Although conductive and convective heat transfer occur, radiant heat transfer is the most significant method of heat exchange in large mammals [22], which is why large mammals, such as cows in a sunny field, seek shade ([23]. Animals will also change their posture or orientation [24] as a means of adjusting the heat received through radiation.

In a building conductive heat transfer will occur through the building envelope, which is why in cold climates buildings are insulated to slow the loss of heat from inside to outside. Convective heat transfer has been a traditional way of cooling buildings in hot climates, by allowing hot air to rise up and vent through a roof opening, drawing in cooler air from near the ground. Radiative heat transfer happens when sunlight enters the building through a window. However, stage one of the literature review did not suggest a useful way of linking examples of thermoregulation in nature to energy efficiency problems in buildings.

### 2.2. Step 2: Classification of Thermal Regulation Strategies in Nature

The second step involved looking for classification systems that might have parallels with thermal regulation mechanisms. The three systems investigated were taxonomy, climate conditions and the different scales at which thermal adaptation occurs (molecule, cell, organism).

The eight major levels of the taxonomic classification are domain, kingdom, phylum, class, order, family, genus and species. Each of these is then split into specific groups. For example, the six main kingdoms in nature are plants, animals, bacteria, archaebacteria, fungi and protozoa. The question for this part of the literature review was whether animals belonging to a particular genus, such as *Panthera* (snow leopard, tiger, jaguar, leopard and lion) used similar thermoregulatory strategies. Given that snow leopards have an enlarged nasal cavity to pre-warm the cold air they breathe in, something that lions living in a much warmer climate do not need, this seemed unlikely and was not pursued further.

When it came to classification by climate it also seemed that animals living in cold climates do not all use the same thermoregulation strategies. For example, reindeer have counter current heat exchange in their legs, polar bears have thick pads and fur on the feet to keep these warm, while other bears hibernate to survive the cold winter.

Classification by scale also failed to produce a way to generalize thermoregulatory strategies. Trees that are dormant in cold winters reduce the water in root tissue to avoid freezing (organism level), but some plants can also secrete anti-freeze proteins (molecular level).

Overall, stage two produced many examples of thermoregulation in nature but did not seem to help in organising the available strategies, so was abandoned and step three started.

### 2.3. Step 3: Thermal Physiology of Heat Regulation in Nature

As a third stage, the thermal physiology of heat regulation was studied in the hope this would create a foundation for categorising thermal adaptation strategies. As explained previously, the themes came from the 479 terms in the UIPS glossary and the many terms on which the experts have agreed were used [8]. The categorised biological thermal adaptation strategies were then used as a means of seeking analogies in architecture. Accordingly, for each mechanism, parallels in energy efficient building design were introduced, and used for the architectural side of the ThBA.

## 3. Results

The results of the three-step literature review are explained below as their findings were connected and together contributed to the development of the ThBA. The stage 2 investigation had provided many examples of thermoregulation, while stage 3 led to structuring and categorisation of the ThBA contents. Even though, step 1 did not directly contribute to the ThBA development it was useful for developing its architectural side.

### 3.1. Main Themes

The themes that emerged from stage 3 of the literature review are outlined below.

#### 3.1.1. Acclimation and Acclimatisation

As suggested by The International Union of Physiological Science (IUPS), acclimation is the physiological and behavioural changes that occur within the bodies of organisms so they can endure environmental stressors. Thermal stressors in this sense are not the familiar aspects of a specific climate but rather a specific climatic factor that has been around for a short period. Acclimatisation, on the other hand, refers to changes that take place in the body during the lifetime of organisms as a response to normal climatic conditions [25].

The meaning of adaptation thus includes both acclimation and acclimatisation, and the changes that occur during both are genotypic and phenotypic adaptation respectively.

#### 3.1.2. Timeframes in Adaptation

According to the IUPS glossary, another important aspect of adaptation is the time interval. The IUPS defines *Crepuscular* and *Nycthemeral* as adaptation activities. The former refers to adaptation strategies that take place at dusk or dawn while the latter indicates these occur on a 24 h basis.

#### 3.1.3. Feedback Loop Control

In the glossary of thermal physiology, the negative-feedback loop forms the backbone of homeostasis, which is the ability of an organism to maintain equilibrium. This enables organisms to respond to environmental stressors in order to stabilise a changing variable. In a system, the comparator compares the received signal (the value of the variable) with a pre-determined set point and the process of adjustment runs until the received signal reaches the set point. In a homeostasis thermal balance mechanism, a sensor monitors the variables [26]. Responses to thermal stressors can be either physiological or behavioural and have been viewed as reflexes [27].

**Figure 2:**
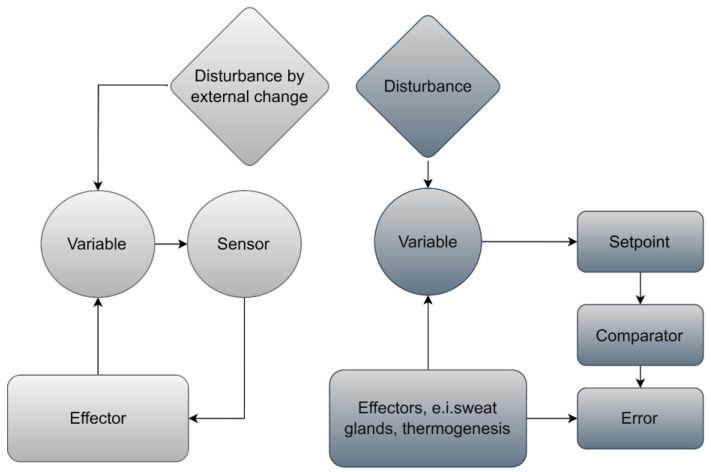
Principles of feedback control in a simple and complex feedback system, adapted from Willmer et al. (2009, p. 34) [28].

Figure 2 illustrates the principles of physiological feedback control in a simple and complex feedback system. The more complex system is associated with autonomic thermal regulation in which the set points detect thermal mismatches.

#### 3.1.4. Autonomic and Behavioural

The IUPS suggested different ways of classifying thermal regulation mechanisms. Some that were obsolete have been replaced by new classification terms. This research used the new terms of autonomic (physiological) and behavioural thermoregulation as they seemed the most appropriate for categorising thermal adaptation strategies.

Autonomic thermoregulation has been defined as the regulation of body temperature through involuntary responses to thermal stressors. Changes in the rates of heat production and heat loss occur through physiological mechanisms such as sweating, shivering, non-shivering thermogenesis and circulatory mechanisms. Behavioural thermoregulation, however, does not happen involuntarily and requires coordinated movement of an organism towards a more favourable thermal environment. The movement in this sense provides the organism with a preferred condition for heat exchange.

According to the IUPS, thermotropism, where an organism such as a plant bends towards a heat source, cannot be recognised as behavioural thermoregulation as the latter is limited to specific behavioural patterns that are controlled by a nervous system.

Another criterion that affected the organisation of the thermal adaptation strategies in the ThBA was the need to reflect the classification of biological organisms as confirmed by the IUPS. Taking this into consideration, the following groupings were deemed important as these were achieved based on the characteristics, limitations and scope of responses of organisms to cold and heat stresses.

#### 3.1.5. Normothermy, Hyperthermia, Hypothermia and Cryothermy

Normothermy, also known as cenothermy and euthermy, describes the conditions where the body temperature is maintained within normal limits. Hyperthermia is a condition where the core temperature is above the range of a species held in a normal state, whereas hypothermia occurs when the core temperature drops below that normal range of body temperature. For each species there is a specified range within which the organism holds a normal active state. Cryothermy refers to the thermal status of a supercooled organism, when the body temperature falls below the freezing point of the body tissue.

#### 3.1.6. Homeothermy, Heterothermy and Poikilothermy

The IUPS glossary divides animals into the two categories of homeotherms—constant body temperature—and poikilotherms—variable body temperature. The two parameters that determine the body temperature are heat gain and heat loss. Homeothermy (homoiothermy) is a thermoregulatory pattern in which temperature variation occurs within defined limits except for conditions where the ambient temperature varies greatly. This means homeotherms regulate their body temperature within a narrow range. This type of thermoregulation happens in tachymetabolic species for which temperature variation takes place during nychthemeral or seasonal cycles. Heterothermy is when the pattern of temperature regulation exceeds the boundary of that of homeothermy. A specific type of the latter is local or regional heterothermy. While the pattern of temperature regulation is the same for heterotherms and poikilotherms, the former occurs only in the thermal shell of homeotherms.

In contrast to homeothermy and the two types of heterothermy, changes in the body temperature of poikilotherms occur over a broad range. This variation happens as a response to ambient temperature fluctuations. While poikilothermy does not include effective autonomic temperature regulation, there are temporary exceptions in some species. For example, several species of flying insects, and large crocodilians and turtles benefit from muscular thermogenesis to maintain their body temperature above the ambient temperature.

#### 3.1.7. Eurythermy and Stenothermy

The IUPS glossary identifies the two distinctive groups of eurythermy and stenothermy. Eurythermy describes the tolerance of organisms to a wide range of environmental temperatures. Heterotherms and poikilotherms belong to this group. Stenothermy is temperature tolerance and happens when the accommodation of an organism to a wider range of temperatures is ineffective. This is the category that includes homeotherms, as the temperature variations in their bodies occur within a narrow range and are not influenced by the fluctuating temperatures in their environments.

#### 3.1.8. Endothermy and Ectothermy

Endothermy and ectothermy divide animals based on the pattern of temperature regulation with a focus on the source of heat on which they are dependent. The skin of ectotherms (commonly called cold-blooded animals) is usually bare, and hence their body temperature tends to follow that of their immediate environment closely. In contrast, the body temperature of endotherms (commonly known as warm-blooded) might not be the same as the surrounding air temperature as they maintain a near constant body temperature. However, the division between ectotherms and endotherms cannot be achieved based on variable or constant body temperatures as a number of ectotherms, such as flying insects, are capable of generating internal heat (Section 3.1.6). This type of heat generation usually takes place through muscular thermogenesis [29].

Ectotherms do not generally generate heat and accordingly, face thermal challenges as they have a very low metabolic rate and do not have the physiological mechanisms to conserve heat. Endotherms use metabolic energy to keep a stable internal body temperature. Endotherms are also capable of adjusting their peripheral blood flow to conserve or dissipate body heat by reducing and increasing the blood flow respectively. Other thermoregulatory mechanisms concern water evaporation from a wet surface. The evaporation takes place if the skin of the animal gets wet either by sweating or spreading saliva (the latter is a habit of kangaroos) [30]. However, this evaporative cooling cannot be used continuously by endotherms as the water from their body has to be replaced, and this is especially critical for small endotherms [31].

Control of heat exchange in endotherms also occurs through physiological responses or physiological adaptation mechanisms. The latter involve strategies in which animals use either their body structures or physiological mechanisms to cope with thermal stresses. These can be viewed as three types, the first being circulatory mechanisms, such as altering blood flow patterns, the second being insulation, such as fur, fat or feathers, and the third evaporative mechanisms, such as panting and sweating [29].

Circulatory mechanisms can be further categorised into two types, these being vasoconstriction and vasodilation and countercurrent heat exchange [32]. In both, the flow of blood is a means of controlling heat gain and heat loss. The former occurs when the blood vessels near the skin surface narrow or expand respectively. Terrestrial animals also use evaporative cooling to lose water from their mouth, skin or nose.

While autonomic (physiological) thermoregulation is unique to endotherms, both endotherms and ectotherms use behavioural thermoregulatory strategies. However, there is a major difference between terrestrial and aquatic animals as the mediums that surround them have different heat transfer coefficients. The air surrounding land-based animals has low thermal conductivity while water permits rapid heat transfer. Relatively, terrestrial animals exploit their environment in order to adjust heat transfer. Davenport [29] states the behavioural adaptation mechanisms used by terrestrial animals to control body temperature (controlling heat gain and heat loss) can be categorised into the four groups of basking, posture, orientation and locomotion. He states animals use specific behaviours to prevent or produce heat and the behavioural means for producing heat can be categorised as clustering and huddling. Behaviours such as shading, migration and burrowing help in avoiding thermal stresses. Increased heat loss is behaviourally achieved by evaporative cooling [29].

#### 3.1.9. Heat and Cold Tolerance

Cold tolerance or cold endurance is defined as “The ability to tolerate low ambient temperatures. This term comprises a variety of physiological properties” [25]. According to IUPS, certain homeotherms are cold tolerant. They are capable of balancing their body temperature when the ambient temperature is low. The relevant mechanisms they use for thermal regulation are either insulation or efficient metabolic heat production. In addition to these, they are able to protect appendages from freezing. For the latter, thermal tolerance is achieved either through vascular control of local heterothermy (protecting appendages from freezing), or general heterothermy such as hibernation. Poikilotherms are also cold tolerant if they can survive low and subfreezing body temperatures (formation of ice-crystals in the state of cryothermy).

Heat tolerance or heat endurance is defined as “The ability to tolerate high ambient temperatures. This term comprises a variety of physiological characteristics” [25]. Homeotherms are often characterized as heat tolerant. They are capable of balancing heat gain and heat loss in locations where the ambient temperature is high. Homeotherms are also considered a heat tolerant species if they can function normally even when their body temperature exceeds their normal range. Selective brain cooling is one of the mechanisms that make this survival possible [33].

In another way of looking at this, thermal adaptation mechanisms and the relative responses to thermal stresses can be approached by avoiding, conforming to or regulating these [34].

Avoiding thermal stress describes the mechanisms animals use to get away from the environment causing the thermal stress through avoidance by movement (e.g., seeking microhabitats such as burrows, or by migration) or avoidance by stopping normal activities (e.g., torpor) [28].

Conforming to thermal stress describes the mechanisms whereby animals undergo changes in their physiological and biochemical levels [35]. Using these mechanisms animals can function though at a very low level. In other words, conforming adaptation mechanisms do not involve huge physiological and biochemical changes but through them the potential damaging effects of a condition such as freezing are avoided [36]. According to the IUPS, thermotolerance or heat shock response (HSR) is a short and rapid action at the molecular level for the purpose of protecting cells and enabling survival for several hours in such a way so that the animal can retain its activity.

Regulating thermal stress requires significant changes in an organism and is a combination of both behaviours (e.g., basking, burrowing, huddling, erecting or concealing appendages) and substantial physiological and chemical transformations. The regulation of thermal stresses is also described as thermal tolerance [28].

### 3.2. Common Aspects of Thermoregulation in Organisms and Buildings

The next stage in the development of the ThBA was to look at the commonalities between thermal regulation in organisms (results of step 3 of the literature review as summarised above) and buildings. In the event, many of these proved inappropriate when it came to structuring the ThBA. However, as they were part of the process they have been briefly summarised below.

The rhythms of organism activities can be either crepuscular or nycthemeral [37]. The former happens only at dawn or dusk while the latter happens anytime during the day. In terms of thermoregulation, behavioural strategies take place on a diurnal basis to enable organisms to respond to the nycthemeral cycle of their core temperature [38].

For a building, variations in the operation of HVAC systems or other thermoregulatory activities such as the way the building occupiers use the space could have nycthemeral patterns. An analogy to crepuscular pattern of thermoregulation could be turning on cooling or heating systems when there are higher temperatures midday and early afternoon and lower temperatures in the early morning and late evening. However, while the analogy of a crepuscular pattern for running space conditioning systems might be valid for houses, it does not seem relevant for an office building or a school, which is less likely to be occupied at dawn and in the evening. This means the building type also governs the rhythms of the thermoregulation.

The concept of feedback loop control is similar for both buildings and the bodies of organisms but remained too general as a factor for organising the ThBA. The parallel to this concept in organisms is the temperature control mechanisms in a building. However, two aspects of the feedback control were relevant for further consideration. For example, the concepts of normothermy, hyperthermia, hypothermia and cryothermy [25] that describe the states of fluctuating body temperature have a parallel in free running buildings, and some energy efficient buildings, for example the zero energy houses at Hockerton [39] are free running. Similarly, homeothermy, heterothermy and poikilothermy [25] were considered applicable to buildings because they pointed to constant or changing body temperature, which could be achieved by running some type of HVAC system. Irrespective of whether the states above describe a range of temperature fluctuations or deviation from the set temperature, connection between the two groupings of normothermy, hyperthermia, hypothermia and cryothermy and homeothermy, heterothermy and poikilothermy emerged. Both groups rely on the mechanism of feedback loop control, which is a mechanism involved in both regulating the temperature in the body of an organism and that of a building.

Likewise, heat and cold tolerance were a potential means of structuring the ThBA. Each category of temperature tolerance had the three sub-branches of thermal adaptation, namely avoiding, conforming to, and regulating thermal stressors. This meant the ThBA could be structured based on the cold and heat endurance capabilities of organisms, broken down into these three sub-branches.

Other similar states to cold and heat tolerance were eurythermy and stenothermy, which are to do with temperature tolerance over a wide range of temperatures. A successful sustainable building in terms of responding to different temperatures and maintaining a satisfactory indoor temperature could be a parallel to eurythermy. Conversely, if the thermoregulatory systems in a building fall short in providing a comfortable environment for the occupants during the highest and lowest ambient temperatures, this is analogous to stenothermy, which is when thermoregulation strategies are only effective over a narrow range of temperatures.

Despite several similarities between thermoregulatory states in organisms and buildings, some thermoregulation concepts in biology cannot be transferred to architecture. For example, acclimation and acclimatisation had no parallel in building design. This is because thermoregulatory strategies in buildings do not evolve over building lifetimes or transfer to their next generations. Nor do buildings tend to move from one climate to another, although the use of portable structures such as tents and how these might be adapted for different climates is a potential link.

Thermal adaptation by definition explains the biological processes that organisms go through to adapt to their environment. However, during these species with poor functions might die while the best fits become prevalent through natural selection. Given this, the concept of distinction and fatality was irrelevant for building design. An imperfect analogy to this concept could be the accumulation of knowledge relating to sustainable building design achieved over the years and the fact that successful strategies would be used for designing the next generation of energy-efficient buildings.

### 3.3. Active and Passive Methods of Thermal Adaptation in Organisms and Buildings

From the thermal physiology terminologies suggested by the IUPS, endothermy and ectothermy emerged as the most appropriate for structuring the ThBA. Endotherms and ectotherms use different sources of heat when it comes to adapting thermally to an environment. The former generate heat within the body, and this has a parallel to running HVAC systems within a building to generate heat. In contrast, ectotherms regulate body temperature through behavioural strategies, such as basking in the sun to raise temperature, or retreating to a shady place to avoid becoming too hot. Parallels in buildings would be raising and lowering window blinds to let the sun in or keep it out. The voluntary (behavioural) and involuntary (autonomic or physiological) aspects of thermoregulation in organisms also seemed useful as a basis for the categorisation of thermoregulation strategies for the architecture side of the ThBA. For endotherms, the control of body temperature mostly takes place within or through organ(s), tissue(s), cell(s) or in other words their physiology, whereas ectotherms control their body temperature mainly through their behaviour. However, there are some exceptions where ectotherms use physiological thermal adaptation strategies to regulate their body temperature.

As a result, the thermal adaptation strategies that come from changes in the physiology of an organism were viewed as active thermoregulatory mechanisms when it came to organising the ThBA. In contrast those strategies that resulted in no change to the body of an organism because all that changed was a behaviour, were termed passive thermoregulatory strategies in the ThBA. For someone designing an energy efficient building if there is some form of change when temperature regulation happens then this is akin to active thermoregulation, whereas if there is no change to the building when there is a thermal change, such a sunlight falling through a fixed window causing a rise in internal temperature, that is akin to passive thermoregulation. Thus, where thermoregulatory strategies take place through circulatory systems or need energy for activation in buildings, they are active, while passive design strategies cause no changes to the building fabric, nor do they make use of HVAC systems. These ideas were embedded in the structure of ThBA. The following sections examine possible active and passive thermal adaptation strategies in organisms and their parallel in buildings.

#### 3.3.1. Active Methods of Thermal Adaptation in Organisms

Penguins and reindeer both use countercurrent heat exchange to avoid excessive heat loss from their extremities. As feet are, relative to the size of the bird or animal, some distance from the body’s core, the arteries taking blood to the feet lose heat to the veins returning blood to the heart, ensuring the blood reaching the feet is cooled.

#### 3.3.2. Active Methods of Thermal Adaptation in Buildings

Buildings that rely on HVAC systems to ensure a comfortable even indoor temperature can be considered as analogous to active thermal adaptation in nature. In some climates in winter one side of the building can be too hot with sun shining through the windows while the other side of the building might be cold. The HVAC system needs to address this by using energy to cool one side and heat the other.

#### 3.3.3. Passive Methods of Thermal Adaptation in Organisms

Behavioural change is a common method of heat regulation in animals. A cat will move into the sun to warm itself and out again when starting to become too hot. Apart from having to expend energy to move the cat remains unchanged. Many animals that live in hot, dry climates such as deserts make use of burrows to avoid midday heat. Using such thermally buffered microclimates is a survival tactic [40].

#### 3.3.4. Passive Methods of Thermal Adaptation in Buildings

Buildings have also been buried or semi-buried in the ground as a passive strategy to reduce heat loss and gain, the Hockerton houses being an example. In earlier times when energy for heating was less readily available, bedrooms were often placed on the east side of the house so as to benefit from the morning sun, and in larger houses the morning or breakfast room would also be placed to receive the morning sun, while the drawing room would benefit from sun later in the day. In such houses, the people would move to make best use of the available energy, but the building did not change.

### 3.4. The ThBA Structure

The analogy between active and passive thermal adaptation strategies in organisms and buildings informed the primary structure of the ThBA, which had three parts. Two were related to passive and active strategies for animals and one for strategies used by plants. The latter had only one part since the main principles relating to active and passive strategies as provided by IUPS did not match properly with the thermoregulatory mechanisms used by plants.

As the aim of this research was to connect the thermal challenges presented by a building design to relevant inspiration(s) in nature, the ThBA needed an additional part for architecture. This meant adding equivalent energy-efficient building design strategies to the other side. Empty boxes were used where there was no parallel design strategy to an existing biological thermal adaptation mechanism. For example, ‘vasodilation’ does not have a parallel in architecture. The absence of an existing parallel was shown by an empty box on the architectural side of the ThBA, although there was no empty box on its biology side.

**Figure 3:**
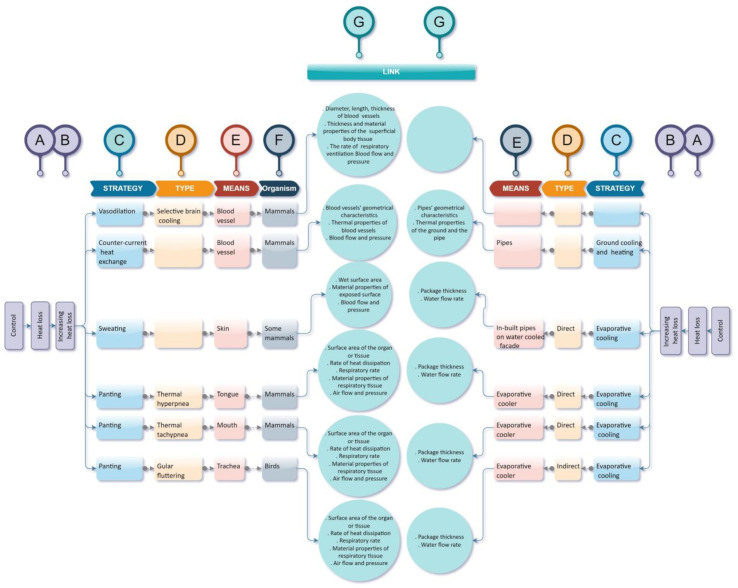
A section of the ThBA related to active strategies for animals, adapted from Imani and Vale [41].

Figure 3 shows a section of the ThBA related to active strategies for animals. Each side of the ThBA consists of different columns. Column A has three main branches called ‘parent actions’ these being heat loss, heat gain and heat generation. As Figure 3 is only part of the ThBA it only shows heat loss strategies. Column B has the three sub-branches of ‘parent actions’, these being increasing heat loss, decreasing heat loss and avoiding heat loss, although only the first part is shown in Figure 3. Column C gives the relevant strategies for each ‘action’ in column B. These strategies then branch into ‘types’ in column D, and for each ‘type’, there is a ‘means’ that refers to the parts of animals or plants responsible for thermal adaptation. Column F gives examples of organisms using each particular strategy.

The architecture side of the ThBA shows sustainable building design strategies presented in the same column types except for column F as the analogy between a building and an organism made column F irrelevant on the architecture side of the ThBA.

In the Design-by-Analogy process, a link should be made between source and target concepts. For cross-disciplinary research, these analogous concepts belong to different fields of knowledge. 

Transferring knowledge from the source design domain into the target design field happens through documenting a series of parameters representing different attributes of the source design concept. The source and target domains were architecture and biology, and the main parameters contributing to each biological thermal adaptation strategy were derived and presented in column G.

Looking at the examples in Figure 3 shows that counter current heat exchange is equivalent to ground cooling and heating and that sweating and panting are related to evaporative cooling in buildings, both direct, as when water is used as the cooling medium in an evaporative cooler (equivalent to sweating), and indirect as when the outdoor air is cooled by a precooled air flow [42] (equivalent to gular fluttering).

Sweating is an autonomic (physiological) mechanism that functions under sympathetic cholinergic and adrenergic control in humans and the animal kingdom respectively. Skin releases water when thermosensitive neurones respond to any changes in the temperature by sending signals to the hypothalamus, which has been described as acting as a set point manager similar to the thermal management system of a building. Panting, which is another analogous strategy to evaporative cooling in buildings can be categorised into three types. In all three, thermoregulation happens through evaporative heat loss as a means of responding to the core temperature overheating. Heat loss is from the mouth area and lungs in mammals while the trachea is the principal organ contributing to gular fluttering in birds.

The links created in the ThBA can be used for translating the thermoregulatory principles in sweating and panting to parallels with the function of evaporative coolers. The ThBA thus lists the key parameters which are necessary for transferring mechanisms from biology to architecture, which then need to be explored by the building designer. For example, in thermal tachypnea the respiratory parameters, air sac properties and cooling efficiency of panting in birds affect their panting mechanism [43]. The designer similarly needs to consider the size and properties of a system of evaporative cooling so that it is suitable for the building. Obviously, the links between evaporative cooling in organisms and buildings are well known, but the hope was that the structure of the ThBA would lead to new opportunities for applying thermoregulatory systems found in organism to buildings. A full description of the creation of the final version of the ThBA and its testing can be found elsewhere [8].

## 4. Conclusions

This article details the development process of a bio-inspired design tool called the thermo-bio-architectural framework (ThBA) for energy efficient building design. The ThBA was developed through conducting a comprehensive literature review on thermal adaptation-associated concepts in biology. These involved exploring different modes of bio-heat transfer, thermal adaptation strategies and the thermal physiology of living organisms. Reviewing these, passive and active ways of thermoregulation linked to the concepts of endothermy and ectothermy emerged as the most appropriate for structuring the ThBA. Heat generation is the key to the concepts of endothermy and ectothermy and the fact that the heat source in nature is either inside or out of the body of organisms, meant there were exact parallels in energy efficient building design. Once created, the ThBA was used in a focus group where biology experts confirmed the inclusiveness, effectiveness and applicability of both the animal and plant parts of the ThBA [41]. This is the first time that a comprehensive framework has been developed to bridge the gap between biology and architecture. The ThBA could assist architects in finding relevant biological thermal adaptation strategies based on the thermal needs of a building.
